# Injectable fillers: current status, physicochemical properties, function mechanism, and perspectives

**DOI:** 10.1039/d3ra04321e

**Published:** 2023-08-10

**Authors:** Jiahong Guo, Wei Fang, Feifei Wang

**Affiliations:** a Yunnan Botanee Bio-technology Group Co., Ltd. Yunnan 650106 China wangfeifei@winona.cn; b Shanghai Jiyan Bio-pharmaceutical Co., Ltd. Shanghai 201702 China; c Yunnan Yunke Characteristic Plant Extraction Laboratory Co., Ltd. Yunnan 650106 China; d Medaesthee (Shanghai) Biotechnology Co., Ltd. Shanghai. 201700 China

## Abstract

With the increasing understanding of the aging process and growing desire for minimally invasive treatments, injectable fillers have great potential for correcting and rejuvenating facial wrinkles/folds and contouring the face. However, considering the increasing availability of multiple soft tissue fillers, it is important to understand their inherent biophysical features and specific mechanism. Thus, in this review, we aim to provide an update on the current injectable filler products and analyze and compare their critical physicochemical properties and function mechanisms for volume-filling. Additionally, future trends and development processes for injectable fillers are also proposed.

## Abstract 2

Injectable fillers have great potential in the correction of facial wrinkles and lines. The longevity of injectable fillers varies from 1 month to 5 years based on the physicochemical properties, fabrication strategy, and function mechanism.

## Graphical abstract

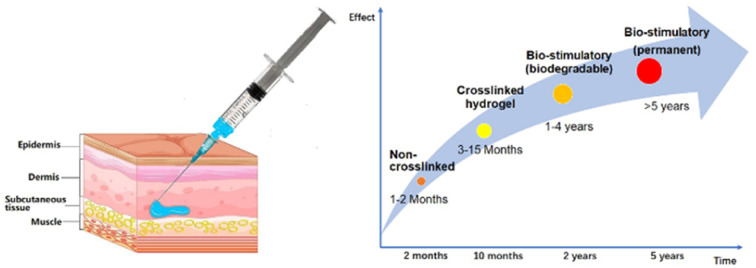

## Introduction

Aging is a complex phenomenon that is influenced by many factors, including genetics, age, diseases, environment, and living habits. Generally, the signs of facial aging include the loss of subcutaneous volume, decrease in elasticity and moisture level in the skin, and an increase in folds and wrinkles. Histologically, this is related to epidermal thinning, dermal atrophy, loss of the elastic tissues within the dermis, and actinic alterations in dermal collagen loss. Consequently, many cosmetic strategies, such as medicine, radiofrequency and implantable biomaterials, have been employed to compensate and correct the signs of aging and restore facial rejuvenation. Among them, injectable fillers have received increasing interest because of their unique characteristics such as easy and minimally invasive procedures.

Injectable fillers have a long history, beginning with autologous fat, liquid paraffin, and silicone oil. However, some of them have been prohibited due to their numerous complications such as hypersensitivity responses and inflammatory reactions leading to ulcerations, fistulas, and skin necrosis. The concept of an ideal filler has been debated for many years, including effective, nonimmunogenic, nontoxic, noncarcinogenic, nonmigratory, easily applied, non-palpable, painless, and long lasting fillers. To date, an increasing number of injectable fillers is emerging and being applied in the commercial market. All injectable fillers are categorized as class III medical devices by regulatory authorities to supervise the medical aesthetics.

Injectable fillers are materials that are injected in or beneath the skin layers to restore the lost volume, smooth lines, soften creases, and enhance facial contours. In general, injectable fillers replenish the lost volume in two ways, *i.e.*, physical filling and stimulating the synthesis of new collagen. The former shows a greater filling effect after injection but disappears gradually. The classical fillers include hyaluronic acid (HA) and collagen, where the latter stimulates collagen formation by creating a space and structure for the entry of fibroblasts and vascular cells, making them more effective in the later phases of injection. The typical fillers are composed of polymer microspheres suspended in solution, such as polylactic acid (PLA), polycaprolactone (PCL), hydroxyapatite (CaHA), and polymethyl methacrylate (PMMA).

Considering characteristics of materials and their mechanisms of action, in this review, we classify polymeric injectable fillers into two categories, *i.e.*, physical fillers and bio-stimulatory fillers. Furthermore, the physical fillers are sub-grouped into HA-based fillers and collagen-based fillers. In addition, we thoroughly analyse the function mechanisms, vital properties, and perspectives of injectable fillers, providing fundamental and constructive suggestions for facial rejuvenation.

## Physical fillers

### Hyaluronic acid (HA)-based fillers

HA, the main component of the extracellular matrix (ECM), is a linear polymer belonging to the class of glycosaminoglycan heteropolysaccharides (GAGs). The structure of HA is composed of repeated disaccharide units of d-glucuronic acid and *N*-acetyl-d-glucosamine linked by alternating β-1,3 and β-1,4 glycosidic bonds.^1^ Endogenously, a human weighing 70 kg has around 15 g of HA, with about half of that contained in the skin.^2^ Accordingly, most injectable filler products are HA based, and presently the most widely utilized. After superficial injection, HA can enhance skin tone and elasticity, while supplements of vitamins, amino acids, and peptides can provide nutritional ingredients to support its positive effects. However, non-crosslinked HA has a limited half-life in the body of around 1–2 days. It degrades rapidly *via* the scission of its glycosidic bonds caused by endogenous hyaluronidase and reactive oxygen species.^3^ Moreover, its elasticity is insufficient to lift tissues, limiting its utility as a filler.

**Fig. 1:**
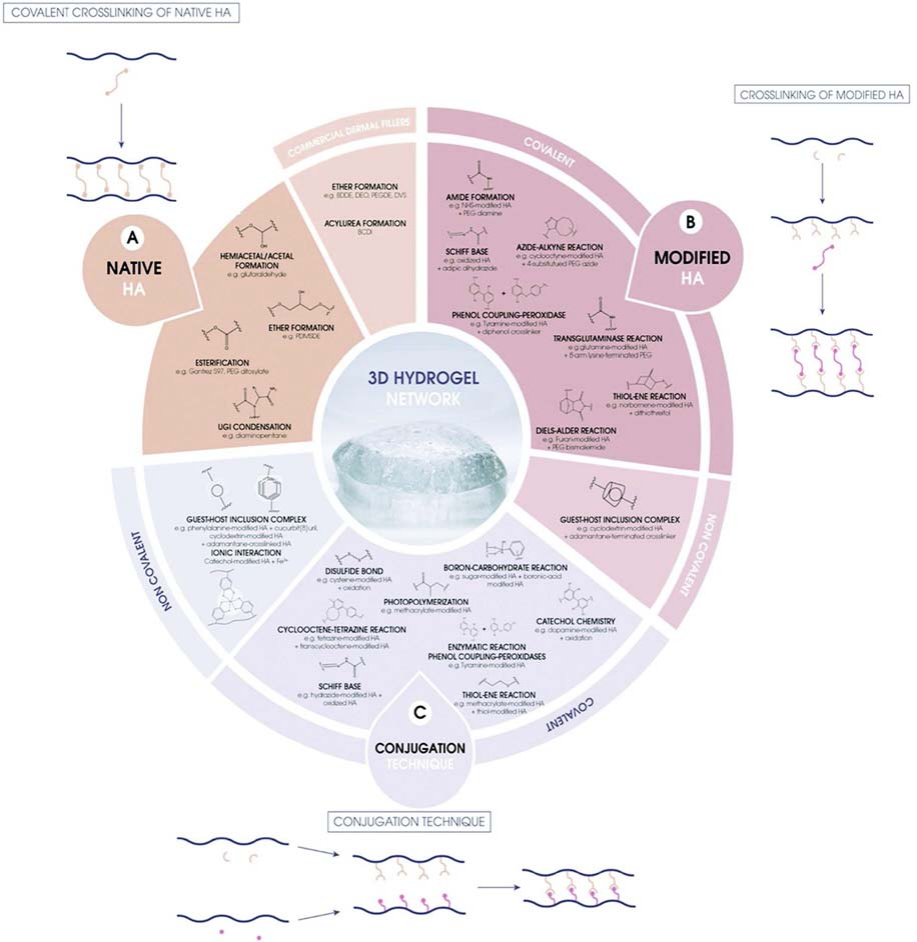
Summary of the strategies for the fabrication of HA hydrogels: (A) starting from native HA and a crosslinker, (B) starting from a modified version of HA and a crosslinker, and (C) using the conjugation technique. This figure has been adapted from ref. 4 with permission from Faivre, Copyright 2021.

Thus, to overcome these flaws, the structure of HA is modified to fabricate gels with a prolonged residence duration and enhanced viscoelastic properties. Faivre *et al.* summarized the multiple chemical routes that can be employed for the preparation of HA hydrogels, as shown in Fig. 1.^4^ Among them, 1,4-butanediol diglycidyl ether (BDDE) and divinyl sulfone (DVS) are the most common crosslinking agents applied in chemical modifications and crosslinking, while the former is more predominate due to its lower toxicity and better reactivity.^5^ The epoxide groups at the two ends of the BDDE molecule preferentially form an ether bond with the most available primary alcohol in the backbone, making BDDE-crosslinked HA fillers durable for up to 1 year. However, considering the mutagenic and carcinogenic potential of BDDE, regulated medical devices require the absence of residual BDDE, and if not, a content considerably below its toxicity threshold of 2 ppm in the final gel.^6^

Presently, multiple commercial HA-based injectable filler products are available, where the main differences in their parameters are their source, concentration, particle size, molecule weight, crosslinking agent, technology for the crosslinking of HA, and the existence of non-crosslinked HA phases. Tables 1 and 2 present the representative HA filler products approved in the United States and China.^7–12^

**Table 1:** Representative HA filler products approved in the United States^a^

Products	Manufacturer	Time of approval by FDA	HA (mg mL^−1^)	Lidocaine	Phase	Needle	Indications	Longevity (M)
Hylaform	Genzyme Biosurgery	April 22, 2004	4.5–6	No	Mono	30G	Injection into mid to deep dermis for correction of moderate to severe facial wrinkles and folds (such as nasolabial folds)	3–4
Hylaform PLUS	Genzyme Biosurgery	April 22, 2004	4.5–6	No	Mono	27G		3–4
Prevelle silk (Hylaform with lidocaine)	Genzyme Biosurgery	February 26, 2008	4.5–6	0.3%	Mono	27G		3–4
Captique	Genzyme Biosurgery	November 2, 2004	4.5–6	No	Mono	27G		3–6
Restylane	Galderma	December 12, 2003; October 11, 2011 for lip augmentation	20	No	Bi	30G	Injection into mid to deep dermis for correction of moderate to severe facial wrinkles and folds (such as nasolabial folds) and for lip augmentation	8–12
Restylane injectable Gel (with lidocaine)	Medicis Aesthetics Holding, Inc	August 30, 2012	20	0.3%	Bi	30G		8–12
Restylane Silk	Valeant Pharmaceuticals North America LLC/Medicis	June 13, 2014	20	0.3%	Bi	30G	Indicated for lip augmentation and dermal implantation for correction of perioral rhytids (wrinkles around the lips)	9–12
Restylane Lyft with lidocaine	Galderma Laboratories	July 1, 2015	20	0.3%	Bi	27G 29G	Injection into mid to deep dermis for correction of moderate to severe facial wrinkles and folds (such as nasolabial folds)	9–12
Restylane Refyne	Galderma	December 9, 2016	20	0.3%	Bi	30G		9–12
Restylane Defyne	Galderma	December 9, 2016	20	0.3%	Bi	27G		9–18
Juvederm ULTRA	Allergan	June 2006	24	No	Mono	30G	Injection into mid to deep dermis for correction of moderate to severe facial wrinkles and folds (such as nasolabial folds)	12
Juvederm ULTRA PLUS	Allergan	June 2006	24	No	Mono	27G		17
Juvederm ULTRA XC	Allergan	January 7, 2010; September 30, 2015 for lip augmentation	24	0.3%	Mono	27G	Injection into mid to deep dermis for correction of moderate to severe facial wrinkles and folds (such as nasolabial folds); injection into the lips and perioral area for lip augmentation	12
Juvederm ULTRA PLUS XC	Allergan	January 7, 2010	24	0.3%	Mono	30G	Injection into mid to deep dermis for correction of moderate to severe facial wrinkles and folds (such as nasolabial folds)	12
Juvederm Voluma	Allergan	October 22, 2013	20	No	Mono	27G	Deep (subcutaneous and/or supraperiosteal) injection for cheek augmentation to correct volume deficit in the mid-face	12–18
Juvederm Voluma XC	Allergan	October 22, 2013	20	0.3%	Mono	27G		
Juvederm Volift	Allergan	2018	17.5	0.3%	Mono	30G	Injection into deep dermis for correction of moderate to severe facial wrinkles and folds (such as nasolabial folds)	6–12
Juvederm Vollure (Volift in Europe)	Allergan	March 17, 2017	17.5	No	Mono	30G	Injection into deep dermis for correction of moderate to severe facial wrinkles and folds (such as nasolabial folds)	6–12
Juvederm Volbella	Allergan	May 31, 2016	15	No	Mono	30G	Injection into the lips for lip augmentation and for correction of perioral rhytids	6–12
Juvederm Volbella XC	Allergan	May 31, 2016	15	0.3%	Mono	30G		
Belotero Balance	Merz Pharmaceuticals	November 14, 2011	22.5	No	Mono	30G	Injection into facial tissue to smooth wrinkles and folds, especially around the nose and mouth (nasolabial folds)	6–10
Elevess (Hydrelle)	Anika Therapeutics	December 20, 2006	28	0.3%	Bi	30G	Use in mid to deep dermis for correction of moderate to severe facial wrinkles and folds (such as nasolabial folds)	9–12

a The crosslinking agent of Hylaform, Hylaform PLUS, Prevelle silk (Hylaform with lidocaine) and Captique is DVS, and the crosslinking agent of Elevess is EDC, and the others is BDDE. The origin of Hylaform, Hylaform PLUS, and Prevelle Silk is rooster comb and the others are non-animal stabilized HA. Mono-phase means the final product only has the crosslinked HA hydrogel and bi-phase means the final product is a mixture of crosslinked HA hydrogel and non-crosslinked HA solution.

**Table 2:** Representative HA filler products approved in China^a^

Products	Manufacturer	Time of approval by NMPA	HA (mg mL^−1^)	Lidocaine	Phase	Needle	Indications	Longevity (M)
Restylane	Galderma	December 15, 2008	20	No	Bi	30G	Injection into the mid dermis for correction of moderate to severe nasolabial folds	8–12
Restylane Perlane	Galderma	June 25, 2018	20	No	Bi	27G or 29G	Injection into the deep dermis and/or subcutaneous tissue for correction of moderate to severe nasolabial folds	9–12
Juverderm Ultra	Allergan	May 29, 2015	24	No	Mono	30G	Injection into mid to deep dermis for correction of moderate nasolabial folds	12
Juverderm Ultra Plus	Allergan	May 29, 2015	24	No	Mono	27G		17
Biohyalux	Bloommage Biotech	September 2, 2019	20	No	Bi	27G or 30G	Injection into mid to deep dermis for correction of moderate nasolabial folds	6–12
Aqua	Bloommage Biotech	March 20, 2020	12	0.3%	Bi	30G	Injection into superficial to mid dermis for correction of frontal wrinkles	6–8
Aqualuna-Mono	Bloommage Biotech	April 28, 2019	20	0.3%	Mono	27G	Injection into mid to deep dermis for correction of moderate nasolabial folds	8
Aqualuna-Bi	Bloommage Biotech	June 19, 2020	20	0.3%	Bi	27G	Injection into mid to deep dermis for correction of moderate nasolabial folds	8–10
Yvoire Classic S	LG Life Sciences, Ltd	July 5, 2013	22	No	Bi	27G or 30G	Injection into the mid to deep dermis for correction of moderate to severe nasolabial folds	6–9
Yvoire Volumes	LG Life Sciences, Ltd	April 25, 2014	22	No	Bi	27G	Injection into the deep dermis to subcutaneous tissue for correction of severe nasolabial folds	8–12
Yvoire Classic Plus	LG Life Sciences, Ltd	March 8, 2016	20	0.3%	Bi	27G or 30G	Injection into the mid to deep dermis for correction of moderate nasolabial folds	6–9
Yvoire Volume Plus	LG Life Sciences, Ltd	December 7, 2015	20	0.3%	Bi	27G	Injection into the deep dermis and/or subcutaneous tissue for correction of moderate to severe nasolabial folds	8–12
Princess Volume	Croma GmbH	May 12, 2017	23	No	Bi	27G	Injection into the mid to deep dermis for correction of nasolabial folds	9
EME	Imeik	February 2, 2021	19	No	Mono	27G	Injection into the superficial to deep dermis for correction of moderate to severe frontal wrinkles and nasolabial wrinkles	12
iFresh	Imeik	December 27, 2019	23	0.3%	Bi	27G	Injection into the mid to deep dermis for correction of moderate to severe nasolabial folds	6–8
Hyalomatrix	Haohai Biotech, lnc.	March 30, 2020	16	No	Mono	27G	Injection into the mid to deep dermis for correction of moderate to severe nasolabial folds	6–12
Matrifill	Haohai Biotech, lnc.	February 8, 2022	16	No	Mono	27G		6–12
Janlane	Haohai Biotech, lnc.	September 8, 2016	20	No	Bi	27G		3–6
Elravie Deep Line	Humedix Co., Ltd	January 26, 2015	23	No	Mono	23G or 27G	Injection into the mid to deep dermis for correction of moderate to severe nasolabial folds	6–8
Elravie Deep Line PLUS	Humedix Co., Ltd	January 26, 2015	23	0.3%	Mono	27G		6–8
Formaderm	Maxigen Biotech, Inc.	April 5, 2016	20	No	Mono	27G or 30G	Injection into the mid dermis for correction of moderate to severe nasolabial folds	6–8
Monalisa	Genoss Co., Ltd	December 12, 2019	24	0.3%	Bi	27G	Injection into the mid to deep dermis for correction of moderate to severe nasolabial folds	8–12
DANAE Line	CG Bio Co., Ltd	March 13, 2019	20	No	Mono	27G		9
ArteFill Universal	Laboratoires Fill-Med Manufacturing S.A.	November 1, 2019	25	0.3%	Mono	27G		18
FACILLE	SciVision Biotech, Inc	April 11, 2014	20	No	Bi	27G		
Refairywave	Funiwei Pharmaceutical	August 12, 2020	18	0.3%	Mono	27G		9
Through young	MBR. Biotech, lnc	June 24, 2019	20 ± 3	No	Mono	27G		9
Filling star	WALOPE.Biotech, lnc	July 15, 2021	20	No	Bi	27G		9
Sofiderm	Professional Manufacturer of Hyaluronic Acid Gel	March 18, 2021	20	No	Mono	26G or 27G	Injection into the mid to deep dermis for correction of moderate to severe nasolabial folds	9
Beatrice	Changzhou Institute of Materia Medica Co., Ltd	March 28, 2018	20 ± 3	No	Mono	30G	Injection into the mid dermis for correction of moderate to severe nasolabial folds	6–9
Singderm	Singclean	January 23, 2020	24	0.3%	Bi	26G or 27G	Injection into the mid to deep dermis for correction of moderate to severe nasolabial folds	9
Belotero Balance Lidocaine	Anteis SA	October 21, 2021	22.5	0.3%	Bi	30G	Injection into the mid dermis for correction of moderate nasolabial folds	6–9

a The origin of these products is non-animal stabilized HA. The crosslinking agent of Hyalomatrix, Matrifill and Refairywave is DVS, and the others is BDDE. Mono-phase means the final product only has the crosslinked HA hydrogel and bi-phase means the final product is a mixture of crosslinked HA hydrogel and non-crosslinked HA solution.

All experimental parameters including HA concentration, molecule weight, particle size, and cross-linking degree impact the physicochemical properties of the product, determining its usage and therapeutic consequences. The crucial physicochemical properties of some products including rheology, cohesivity, extrusion force and swelling factor are summarized in Table 3.

**Table 3:** The physicochemical properties of partial HA filler products^a^

Products	HA (mg mL^−1^)	*G*′ (Pa)^13^	*G*′′ (Pa)	Tan *δ*	SwF (mL g^−1^)^14^	Dw (mg)^8^	Injection force (*N*)^15^
Restylane	20	349	145	0.42	2.8	15	8
Restylane Perlane	20	411	199	0.48	2.6	14	16
Restylane Silk	20	344	79	0.23	2.7	18	10
Restylane Lyft with lidocaine	20	545	69	0.13	2.8	14	21
Restylane Refyne	20	47	7	0.16	9.7	28	6
Restylane Defyne	20	260	16	0.06	6.4	22	8
Juvederm ULTRA	24	76	18	0.23	9.5	29	8
Juvederm ULTRA PLUS	24	148	24	0.16	8.3	27	14
Juvederm Voluma	20	284	58	0.21	5.7	20	25
Juvederm Voluma XC	20	307	29	0.09	4.8	18	20
Juvederm Volift	17.5	179	42	0.23	7.2	24	10
Juvederm Vollure (Volift in Europe)	17.5	273	32	0.12	4.1	16	16
Juvederm Volbella	15	159	21	0.13	3.8	15	8
Belotero Balance	22.5	41	19	0.47	16.9	48	6
Yvoire Classic S	22	286	103	0.36	5.4	No data	9.8
Yvoire Volumes	22	253	73	0.29	5.9		12.7
DANAE Line	20	260	100	0.39	5.9		19
Elravie Deep Line	23	159	39	0.25	7.1		25
Elravie Deep Line PLUS	23	198	59	0.3	6.9		24.5

a The HA concentration is given in accordance with the product instructions.

The rheology property of a product is correlated with the flow and deformation of its materials in response to stress. The elastic modulus (*G*′), which is also known as the storage modulus, measures the hardness of a material and its capacity to resist deformation. *G*′ represents numerous factors that affect gel strength, and thus has become a major parameter to differentiate products. The viscous modulus (*G*′′), which is also called the loss modulus, refers to the energy dissipated by friction throughout a stress cycle and represents the failure of a gel to entirely recover its shape after the shear force is removed. In terms of rheologic tailoring, firmer gels with a greater *G*′ are more resistant to deformation but may feel lumpier after implant and produce more adverse effects such as pain, inflammation, and edema. Thus, products with a high *G*′ are employed for contouring and sculpting in deeper areas, such as the chin. Conversely, fillers with a lower *G*′ are softer and provide a more natural feel upon implantation, making them more suitable for the treatment of soft tissue and superficial zones such as the lip, glabella, and periorbital area. Intermediate *G*′ fillers can be utilized for dynamic wrinkle correction, as well as supporting and contouring in regions of facial animation, such as the midface.^13,16^ Furthermore, the addition of lidocaine can significantly minimize the discomfort to patients during injection without substantially affecting the rheologic properties of the fillers.

**Fig. 2:**
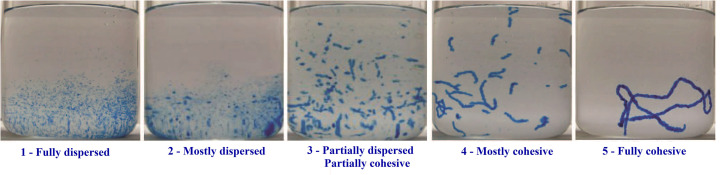
Examples of cohesivity scores. Cohesivity of three different crosslinked HA fillers and their corresponding cohesivity. This figure has been adapted from ref. 17 with permission from Hema, Copyright 2015.

Cohesivity characterizes the internal adhesion forces between the cross-linked HA particles that remain intact and not dissociated under an external force. Based on photographs, the five principal patterns of gel behavior are listed in Fig. 2.^17^ Usually, the cohesivity of a hydrogel is calculated based on the average drop in its weight in a syringe under stress.^8^ It has been reported that products with a higher cohesivity have higher integration and lift capacity. Low-cohesivity fillers are generally recommended for modest rhytid correction because they are easier to shape and disperse uniformly in the skin. Fillers with high cohesivity are more suitable for re-volumizing larger areas of loss.^18^ A dermal filler must be cohesive enough to withstand compression forces after injection, avoiding its migration.^19^

The extrusion force is the force exerted by HA gels injected through a syringe needle with a specific size, which is determined by *G*′, particle size and distribution range. Currently, there are no international standards or methods to govern the injection of HA gels; however, various syringeability and injectability tests can be found in the literature.^15,20,21^ Gels with larger particles or *G*′ will be more difficult to inject through a small-bore needle. In the extrusion process, a limited range of particle size distribution can reduce interruptions and fluctuations in force. To facilitate particle extrusion, stiff gels with a greater *G*′ must be designed as smaller particles with a narrower particle size range or incorporation of a tiny quantity of non-crosslinked HA chains to lubricate and fluidize the gel. Softer gels with a low *G*′ can have a wider particle size range and can be easily distorted as they pass through the needle.^22^

The swelling factor, which is also known as the gel fluid absorption, characterizes the capacity of a gel to expand as it binds water, while remaining a single phase *in vitro*. The swelling factor was evaluated by thoroughly mixing 0.5 g of gel with 6 to 8 mL of saline, which then increased to 10 mL.^14^ When the swelling factor is close to equilibrium, a gel will not swell significantly after injection; otherwise, it will rapidly absorb water from the surrounding tissue fluid. The swelling factor varies between products and is affected by the concentration of HA and the physical limits imposed by crosslinking, where *G*′ increases and the swelling factor decreases as the degree of crosslinking increases.

**Fig. 3:**
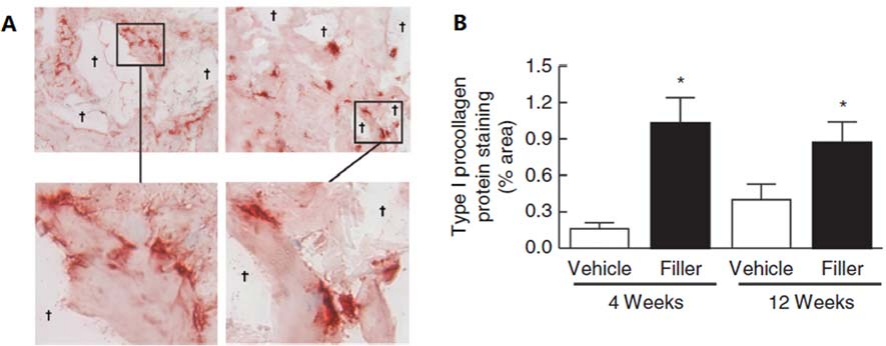
(A) Immunostaining of type I procollagen protein at 4 weeks (left panel) and 12 weeks (right panel). Inset display elongated morphology of immunostained fibroblasts adjacent to the filler. (B) The level of type I procollagen protein. This figure has been adapted from ref. 23 with permission from Quan, Copyright 2013.

Crosslinked HA fillers are thought to volumize soft tissues due to their water-binding and space-filling characteristics. Many studies have proven that HA injectable fillers provide structural support for the extracellular matrix (ECM) in the dermis, consequently stimulating fibroblast activation and collagen synthesis. Quan *et al.* injected crosslinked HA fillers into aged skin and discovered that the fibroblasts around the filler had elongated morphologies, indicating the enhanced mechanical forces and structural support within the dermal ECM (Fig. 3). Importantly, fibroblast elongation is associated with the upregulation of the TGF-β signaling pathway and its downstream targets CTGF/CCN2 and type I procollagen.^23^ Another study suggested that HA promotes MAPK/ERK phosphorylation and TGF-β1-dependent fibroblast proliferation by facilitating interactions between CD44 and EGFR.^24^ Thus, the effects of hydration, direct volume filling, and new collagen production appear to represent the results of soft tissue augmentation using crosslinked HA fillers.

Crosslinked HA filler may elicit allergic symptoms such as temporary erythema, edema, itching, and moderate swelling. Furthermore, due to the anti-coagulant activity of HA and the Tyndall effect, it may occasionally result in the formation of superficial bruises, pale nodules, hypertrophic scars, and tiny discolored regions. Serious injuries have also been documented, including foreign-body granulomas and vascular occlusion.

The most prevalent possible consequences are immune-mediated adverse events and inadvertent injection, which tend to resolve spontaneously within a few hours or, at most, a few days. In this case, a skin allergy test performed before treatment can largely prevent foreign-body granulomas.^25^ Rapid enzymatic degradation with the appropriate number of hyaluronidases can alleviate vascular occlusions.^26^ Hylenex, an FDA-approved human recombinant formulation of hyaluronidases, promotes HA elimination through the kidneys with its effects lasting around 48 hours.^27^ Furthermore, these risks can be minimized with a masterful understanding of the facial vascular anatomy. It may also be avoided by the expertise of cosmetic treatment professionals.

### Collagen fillers

Collagen is the main component of the ECM in the human body, providing physical support, great tensile strength, and resilience to tissues and organs. Besides, collagen interacts with a number of macromolecules, including integrins, decorin, fibronectin, heparin, and matrix metalloproteases (MMPs) to regulate critical functions during tissue regeneration. Aging is accompanied by the loss, disorganization and fragmentation of collagen. The fragmentation of collagen fibers impairs its interaction with fibroblasts, changing the cell morphology, and thereby reducing the mechanical forces.^28,29^ Thus, collagen fillers can be utilized to replenish collagen, correct the loss of volume, and maintain the health of the ECM environment. To date, several types of collagen products have been approved to act as soft tissue fillers. Table 4 reviews the currently approved collagen products.

**Table 4:** Representative collagen fillers approved in the United States

Product	Year of approval by the FDA	Collagen origin	Composition	Crosslinking agent	Skin test	Storage	Indication	Duration
Zyderm I	1981	Bovine collagen	3.5% bovine collagen, 0.3% lidocaine	No	Yes	Refrigeration	Injection into the superficial papillary dermis for correction of superficial lines and wrinkles, as well as acne scars	3–4 m
Zyderm II	1983		6.5% bovine collagen, 0.3% lidocaine	No	Yes	Refrigeration	Injection into the papillary dermis to treat lines, wrinkles, traumatic scars	3–4 m
Zyplast	1985		3.5% bovine crosslinked collagen, 0.3% lidocaine	Glutaraldehyde	Yes	Refrigeration	Correction for deeper lines and folds such as mid-deep dermis and nasolabial fold	4–6 m
CosmoDerm I	2003	Human-derived collagen	3.5% human-derived collagen, 0.3% lidocaine	No	No	Refrigeration	Injection into the superficial papillary dermis for correction of soft tissue contour deficiencies, such as wrinkles, folds and scars	3–6 m
CosmoDerm II	2003		6.5% human-derived collagen, 0.3% lidocaine	No	No	Refrigeration		3–6 m
CosmoPlast	2003		3.5% human-derived crosslinked collagen, 0.3% lidocaine	Glutaraldehyde	No	Refrigeration	Injection into the mid to deep dermis for correction of soft tissue contour deficiencies, such as wrinkles, folds and scars	4–7 m
Fascian	1999	Human cadaveric collagen	Human fascia gastrocnemius derivatives, 0.5% lidocaine	No	No	Room temperature	Correction for wrinkles, scars, fat atrophy, diffuse depressions, paralyzed lips and tongues, and nasolabial folds	8–12 m
Dermologen	1990		Human cadaveric collagen and matrix material (elastin, GAGs)	No	No	Limited	Correction of nasolabial folds, lip wrinkles, acne, and deep pitting	Not known
Cymetra	2000		Human cadaveric dermal matrix, 1% lidocaine	No	No	Limited	Correction of nasolabial and radial folds, depressed corners of the mouth, lip augmentation and scarring.	3–6 m
Isologen	1999	Patients' autologous collagen	Autologous collagen	No	No	Refrigeration	Correction of moderate to severe nasolabial fold wrinkles	Not known
Fibrel	1989	Porcine-derived collagen	Porcine-derived collagen	No	Yes	Refrigeration	Correction of depressed cutaneous scars, facial lines and wrinkles	3–4 m
Evolence	2008		Porcine-derived crosslinked collagen	d-ribose	No	Recalled	Correct the moderated to severe facial wrinkles and folds	4–6 m

Collagen filler was initially applied in clinic in 1951, while Zyderm I was the first FDA-approved collagen filler product in 1981. Following that, Zyderm II and Zyplast emerged successively.^30,31^ However, because the collagen in these products is sourced from bovines, intradermal skin allergy testing is necessary before the injection operation. After two skin tests, the allergy risk can be lowered from 3% to 0.5%.^32^

Subsequently, aiming to decrease the potential immunogenicity, human-derived collagen products were developed, which are known as CosmoDerm I, CosmoDerm II and CosmoPlast. Their specific parameters are similar to that of their counterparts Zyderm I, Zyderm II and Zyplast. Allergy testing is not necessary when injecting these implants because immunogenicity studies have demonstrated a significant decrease in potential hypersensitivity reactions (less than 1.3%).^33^ Dermologen, Cymetra and Fascian are produced from collagen fibers and extracellular matrix derived from human cadaveric tissue. The procedures used to acquire these tissues include cell rupture and removal, prion inactivation, two viral inactivation phases, and lastly sterilization.^34,35^

Isologen is a collagen product that is created as an autologous implant derived from the skin of the patients. A 3 mm punch biopsy is normally collected from the area behind their ear and delivered to isologen for culture. After weeks of cultivation, 1 to 1.5 cm^3^ of fibroblasts and extracellular matrix components are transported back to the physician for injection.^36–38^ Isologen has no risk of infectious agent transmission from an animal or human donor, and it does not cause hypersensitivity to foreign proteins. However, it requires several procedures and is very expensive.

In addition to bovine collagen, porcine-derived collagen was employed as a filler product. Fibrel, a lyophilized version of gelatin powder, was authorized by the FDA in 1989 for the treatment of depressed cutaneous scars and for facial lines and wrinkles in 1991. Evolence, which is crosslinked by d-ribose, was CE marked in 2004 and approved by the FDA in 2008 to correct moderate to severe facial wrinkles and folds.

The most significant disadvantage of collagen products in comparison to HA injectable fillers is their immunogenicity. Although telopeptide removal and crosslinking methods can be used to enhance the immunogenicity of collagen, the risk of viral infection remains a challenge because the tolerance of collagen products to terminal sterilization is limited. Accordingly, recombinant human collagen-based products have become the focus of research. Recombinant human collagen (rhCollagen), which is identical in structure and functionality to human collagen, was successfully produced by expressing a particular gene segment transcribed into the host.^39^ Unlike tissue extract protein, rhCollagen is not immunogenic and not allergic, and it has an intact triple helix structure that demonstrates superior biological function. Ma *et al.* synthesized a potential hydrogel based on human-like collagen and chitosan, which can be employed as a dermal filler with a less intense inflammatory response in the presence of dialdehyde starch.^40^ Seror *et al.* created photocurable rhCollagen by chemically modifying the protein to facilitate crosslinking under illumination, which was used as a dermal filler and a bioink for 3D-printed breast implants.^41^ Karisma Face RhCollagen is a newly introduced injectable soft filler that is comprised of R polypeptide α1 chains of type I collagen, high molecular weight HA and carboxymethyl cellulose (CMC). It restores the firmness and structure of the skin gradually, visibly, and permanently.

## Bio-stimulatory fillers

**Fig. 4:**
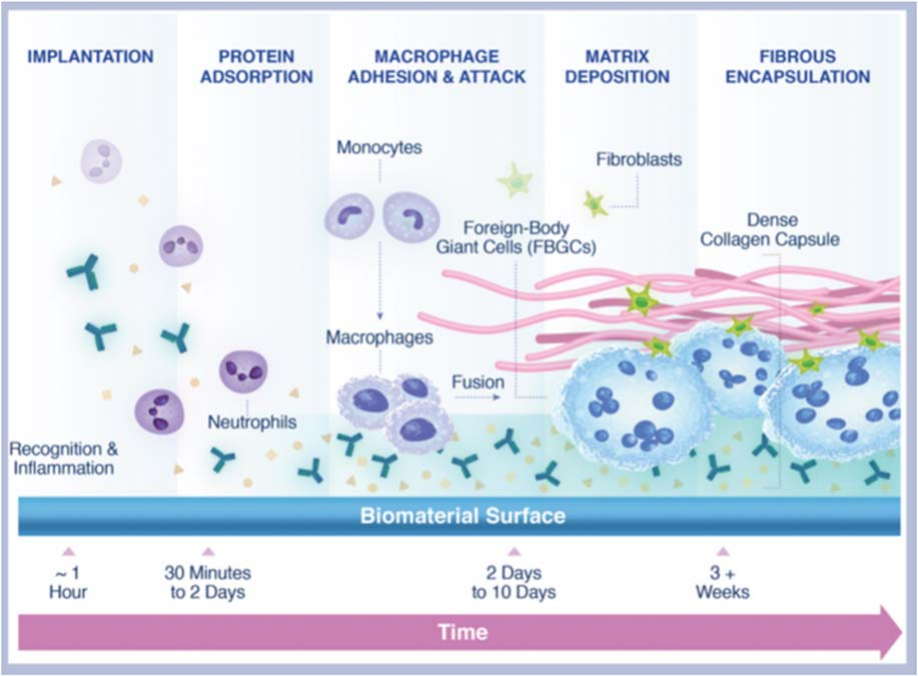
Foreign body reaction to a biomaterial. This figure has been adapted from ref. 42 with permission from Fitzgerald, Copyright 2018.

Protein absorption, cell recruitment, and fibrotic encapsulation are the three sequential stages in the foreign body response to foreign material. Once an implant is injected, microspheres evoke a subclinical foreign body inflammatory response, culminating in the encapsulation of the microparticles, followed by fibroplasia and collagen type I deposition in the extracellular matrix^42^ (Fig. 4). Based on this, some biomaterials such as PLLA, PCL, PMMA, and CaHA have been employed as collagen stimulators to augment soft tissue volume.

### Poly(l-lactic acid) (PLLA)

Poly-l-lactic acid is a biodegradable, biocompatible and synthetic polymer invented by a French chemist in 1954. It has been employed as a resorbable suture material and plates and screws in orthopedic, neurologic, and craniofacial surgeries. Unlike other fillers that provide instant correction, the volume of the injected regions increases after injection due to the mechanical distention from the suspension of the microspheres, followed by new collagen production *via* fibroblast activation by the PLLA microspheres. Specifically, macrophages and foreign body giant cells detect PLLA as a foreign body and recruit and stimulate fibroblasts *via* TGFβ1 to proliferate and differentiate into myofibroblasts. (Myo-)fibroblasts encapsulate PLLA particles with collagen type III and deposit fibrotic collagen type I around the capsule.^43^ Importantly, the foreign body response is biocompatible, where PLLA microspheres are degraded slowly to carbon dioxide and water over a period of weeks to months.^44^ PLLA microspheres were destroyed completely over the course of 9 months, with no leftover PLLA or scarring fibrosis observed.^45^ Sustained volumetric expansion and correction with PLLA have been reported for up to 2 years, although the majority of PLLA would have been entirely metabolized by that time.^46^

Sculptra is composed of lyophilized PLLA particles, which was authorized by the FDA for the recovery and correction of adipose atrophy in HIV patients in 2004. Sculptra aesthetic was approved in 2009 for grid injection into the deep dermis to treat moderate to severe nasolabial folds and other wrinkles/folds. Each Sculptra syringe has 150 mg PLLA microspheres and 217.5 mg sodium carboxy-methylcellulose and mannitol. Before injection, PLLA powders must be reconstituted 24 to 72 h to fully hydrate and form a very viscous hydrogel. The vial should be vigorously shaken just before injection to ensure homogenous mixing.^47^ Löviselle, which was approved by NMPA in 2021, consisted of mixed freeze-dried PLLA microspheres powder, mannitol, and sodium carboxymethyl cellulose. These freeze-dried powders need to be dissolved with 0.9% sodium chloride before use.

**Fig. 5:**
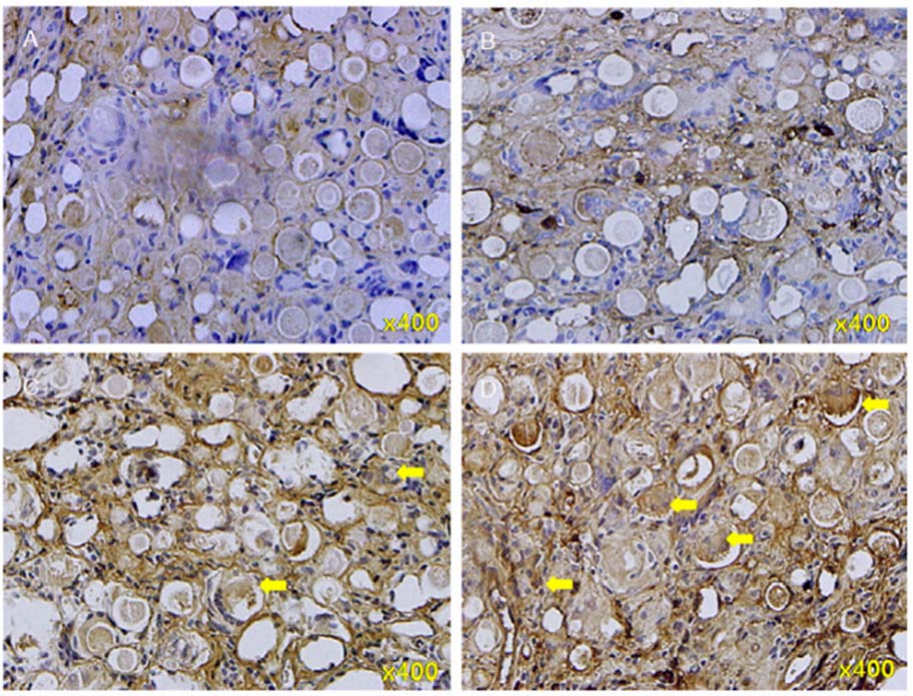
Immunohistochemical staining of type I collagen in neo-tissues in the 4th to 20th week after poly-d, l-lactic acid filler injections. (A) 4th week; (B) 8th week; (C) 12th week; and (D) 20th week. Yellow arrows: type I collagen, with increasing number from A to D. This figure has been adapted from ref. 48 with permission from Lin, Copyright 2019.

PDLLA (poly-d,l-lactic acid) is also a biocompatible, biodegradable, bio-stimulatory and long-lasting material that can be used as a new subdermal stimulatory filler. AestheFill is a filler composed of 30 to 70 μm PDLLA microspheres suspended in sodium carboxymethylcellulose. Lin *et al.* injected AestheFill into SD rats and identified extracellular type I collagen in areas between and on the outside surfaces of the microspheres after 4 weeks and inside the individual microspheres by 20 weeks (Fig. 5).^48^

The short-term adverse effects following the injection of PLLA and PDLA microspheres include moderate transitory localized erythema, ecchymosis, and edema, as predicted. Nonvisible, palpable subcutaneous nodules, which typically disappear spontaneously, are some of the long-term adverse effects, together with chronic granulomatous responses (0.2–1.2% incidence).^49^

### Polycaprolactone (PCL)

Poly-caprolactone (PCL) is a biocompatible, biodegradable, and bioresorbable polymer widely used in surgical sutures, artificial blood vessels/skin, bone and soft tissue fillers, and tissue scaffolds. Similar to PLLA, the degradation end products of PCL are CO_2_ and H_2_O, which can be totally eliminated from the body.^50^

Ellanse, which consists of 30% PCL microspheres and 70% carboxymethylcellulose (CMC) gel carrier, presents different functions. The CMC gel is responsible for the immediate impact due to the injected volume filling capacity and very hygroscopic feature of CMC, but it is resorbed within 2–3 months. The PCL microspheres have a long-lasting impact due to the synthesis of collagen and scaffold formation. Based on the chain length (molecular weight) of the initial PCL chains within the microspheres, Ellanse is categorized into four models with durations ranging from 1 year to 4 years.

**Fig. 6:**
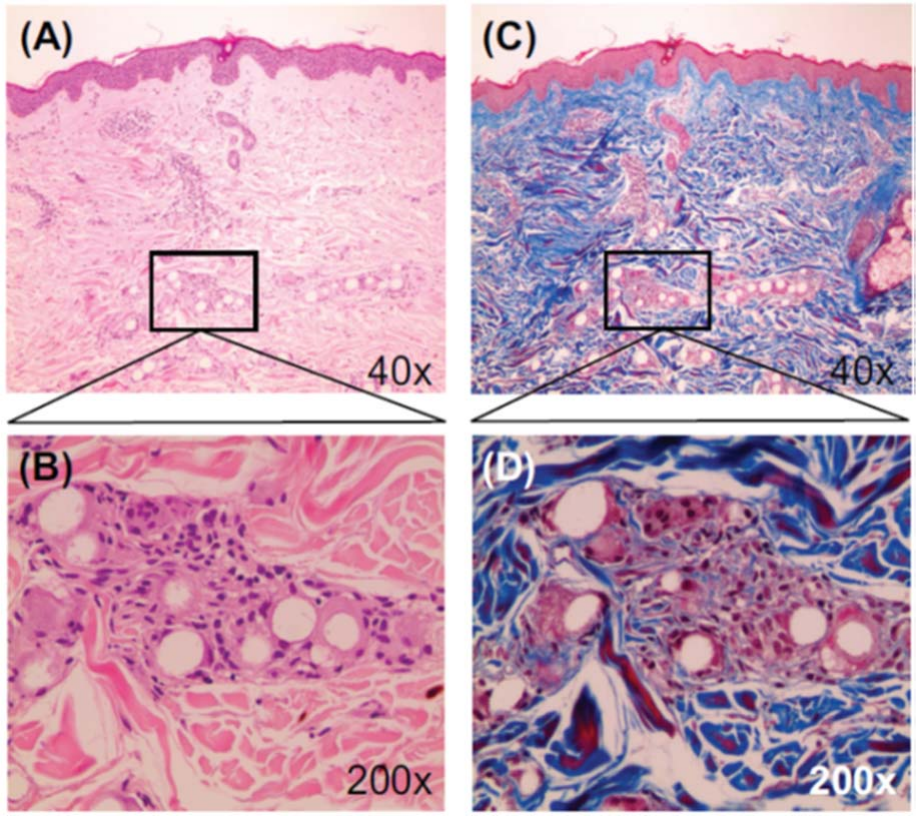
Microscopic images (13 months post-injection) showing PCL microspheres surrounded with collagen deposition and a mild fibroblastic and histiocytic tissue response. Stains were H&E (A and B) and MT (C and D). This figure has been adapted from ref. 51 with permission from Kim, Copyright 2014.

**Fig. 7:**
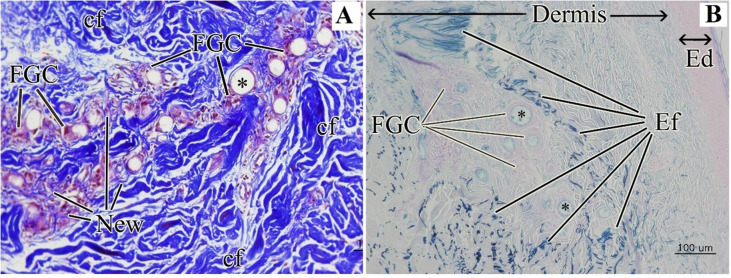
(A) Dermis with Masson trichrome stain and collagen fibers stained blue (4 years post-treatment). (B) Dermis after PCL injection with special stain (4 years post-treatment). Elastin fiber stained black in Verhoeff's Van Gieson (EVG) stain and blue in Victoria Blue (VB) stain. FGC: foreign-body giant cells; cf: collagen fiber; new: new collagen fiber; Ef: elastic fiber; and Ed: epidermis. This figure has been adapted from ref. 52 with permission from Kim, Copyright 2019.

Kim *et al.* confirmed the dual-effect of EllanséTM-M, *i.e.*, direct and “delayed” volumizing effect. According to human biopsies at 13 months, new collagen formed around the PCL particles through the activation of neocollagenesis (Fig. 6).^51^ Kim further evidenced the collagen production and skin texture improvement in the human temple after treatment with Ellansé-M. According to biopsies, new collagen fibers, elastic fibers, and neovascularization with new capillaries were observed at 1 year and 4 year post-treatment^52^ (Fig. 7).

**Fig. 8:**
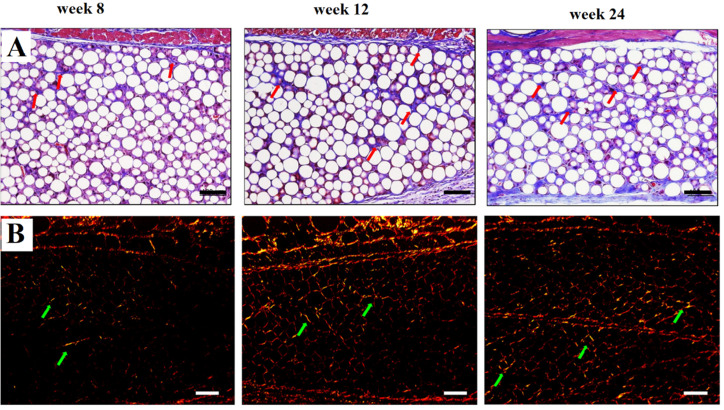
Histological response to Ellanse fillers after injection, stained by Masson's trichrome (A) and Picrosirius red (B). This figure has been adapted from ref. 53 with permission from Oh, Copyright 2022.

To further identify the type and content of collagen synthesis, Oh *et al.* used Masson's trichrome (MT) and Sirius red (SR) staining. MT staining revealed that the number of nuclei increased in both groups as the content of collagen increased following fibroblast stimulation by the microspheres. SR demonstrated that type III collagen (thin green and yellow color) was freshly generated after 8 weeks of injection, and thicker and mature red and reddish-yellow type I collagen fibers were created over time (Fig. 8).^53^

Globally, Ellanse has been shown to have no serious adverse effects, granuloma or vascular complications, with only a few initial injection-related responses, mostly edema or ecchymosis, which are generally minor and resolve naturally without intervention within a few days.

### Calcium hydroxylapatite (CaHA)

Calcium hydroxylapatite (CaHA) is a component of human bone and teeth, and synthetic CaHA has been utilized in medicine as a biodegradable and biocompatible substance for more than 20 years. CaHA is the main ingredient in Radiesse to treat moderate to severe facial wrinkles and folds. Each Radiesse syringe consists of 30% CaHA microspheres suspended in 70% carrier solution including sterile water, glycerin, and carboxymethylcellulose. Radiesse has double effects, including immediate mechanical filling of the carrier solution and long-term neocollagenesis through the CaHA microspheres. Between them, the immediate filling effect will be absorbed with time and replaced by collagen due to the stimulation of the CaHA microspheres.

**Fig. 9:**
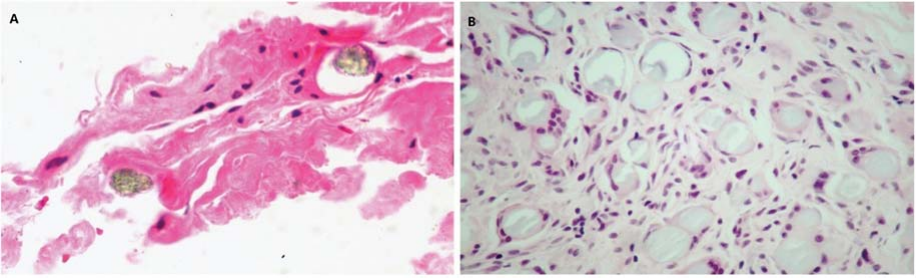
Tissue biopsies following injection with Radiesse (A) 3 weeks and (B) 6 months post-injection. This figure has been adapted from ref. 56 with permission from Lorenc, Copyright 2018.

After injection, the deposited CaHA particles can mimic the host environment and support the ingrowth of fibroblasts and collagen. The filling-up period is up to 2 years due to the slow enzymatic metabolism and phagocytosis of the CaHA microspheres.^54^ The CaHA microspheres are eventually totally degraded into calcium and phosphate ions and secreted by the body, following the same metabolic process as the bone debris produced by typical bone fractures.^55^ Lorenc *et al.* evaluated the fibroblastic response of Radiesse and discovered that fibroblasts surrounded the CaHA microspheres after 3 weeks, and 6 months later, the microspheres varied in size when they are broken up by the fibrous connective tissue reaction and surrounded by fibrous connective tissue (Fig. 9).^56^

Radiesse does not require skin testing because it is immunologically inactive. However, its adverse events are the same as other semi-permanent fillers such as PLLA and PCL microspheres. Its short-term side effects include moderate, temporary localized erythema, ecchymosis, and edema. Its long-term adverse effects include nodules and granulomas, particularly when injected into the lip. Broder found that Radiesse dissipates soon after injecting into the lips and the CaHA particles cluster together, generating noticeable hard white nodules in the lip.^57^

### Poly(methyl methacrylate) (PMMA)

Polymethylmethacrylate has been used successfully in medical implants such as orthopaedic bone cement and craniectomy plates for more than 65 years. Compared with other biomaterials for the treatment of wrinkle lines and soft tissue loss, which degrade within a few months to years, PMMA is a non-resorbable synthetic chemical with a permanent effect.^58^

Artecoll is a permanent filler made up of 20% PMMA microspheres suspended in an 80% bovine collagen solution (3.5% bovine collagen, 2.7% phosphate buffer, 0.9% sodium chloride, 0.3% lidocaine hydrochloride and 92.6% water).^59^ ArteFill is another version of Artecoll that was rebranded as Bellafill in 2014.^60,61^ After deep dermal injection of these products, the collagen carrier is degraded by the body and totally replaced by the its own collagen at the same pace, resulting in consistent augmentation. The permanent PMMA microspheres can be considered as “living implants” that provide tissue augmentation through fibroplasia with a 5 year lasting effect.

**Fig. 10:**
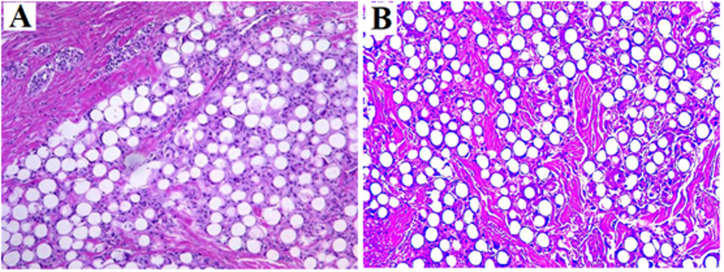
(A) Histology of ArteFill at 3 months, where capillaries have infiltrated the implant, which has become the patient's own tissue. (B) Histology of ArteFill at 10 years, with mature connective tissue including active fibroblasts, microencapsulation of each microsphere, capillary ingrowth, and little foreign body reaction. This figure has been adapted from ref. 60 with permission from Lemperle, Copyright 2010.

ArteFill (Bellafill) has also been evaluated for its effectiveness and safety in the correction of atrophic facial acne scars. In a double-blind, randomized, multi-center, control study, 64% of the ArteFill-treated participants and 33% of control (saline) subjects were successful.^62^ Lemperle *et al.* further revealed that all the PMMA microspheres are entirely encapsulated and surrounded by fibroblasts and collagen fibers, with only a few macrophages at 3 months. Strong bands of mature collagen fibers with fully intact capillary vasculature could still be seen surrounding the intact PMMA microspheres after 10 years (Fig. 10).^60^

Its short-term side effects include expected mild, temporary localized erythema, ecchymosis, and edema. The predominant long-term adverse event documented in a 5 year safety and satisfaction study evaluating the use of Bellafill in the treatment of nasolabial folds is the formation of granulomas, which had an overall incidence rate of 1.7%.^63^

**Fig. 11:**
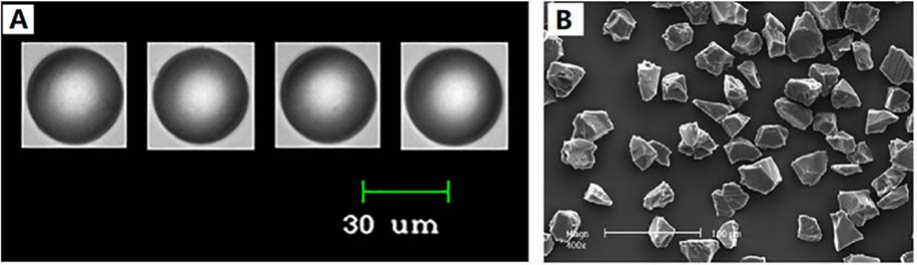
(A) Microspheres of Bellafill, showing uniformity of size and shape. (B), microspheres of Metacrill, showing the irregular shape of the particles. This figure has been adapted from ref. 61 with permission from Gold, Copyright 2018.

Furthermore, PMMA is a key component in Metacrill and NewPlastic. In South America, Metacrill, a soft tissue filler composed of PMMA microparticles suspended in a carboxymethylcellulose colloid, has been utilized to treat facial rhytids, acne scars and facial herniatrophy.^64^ The microparticles range in size from 1 to 80 μm and have an uneven shape (Fig. 11).^61,65^ NewPlastic consists of PMMA particles suspended in sodium hyaluronate (2%), D-1 propanediol (10%), and a pyrogenous solution.^66,67^ The microspheres of NewPlastic are 30–70 μm in size and non-spherical, with conjoined particles. Thus, due to the particle size, morphology, and surface characteristics of these two products, there are not as popular as Bellafill.

For bio-stimulatory injectable fillers, several key parameters such as the size and morphology of the particles, the viscosity of the carrier gel and rheology property influence their longevity, inflammatory, and function.

When the diameter of a microsphere is in the range of 15–20 μm, it is at risk of being phagocytosed by macrophages, resulting in giant cell formation and granulomatous inflammation.^60^ Microspheres with diameters smaller than 10 μm have also been demonstrated to promote lymphocyte infiltration and vascularization. Lemperle *et al.* reported that the smaller the microspheres (to the threshold of phagocytosis), the larger their combined surface area in a given volume and the greater the total amount of new collagen formation. Microspheres with a mean diameter of 100 μm stimulate only about 56% connective tissue, whereas microspheres with a mean diameter of 40 μm promote around 80% connective tissue ingrowth.^68^ However, oversizing of the microspheres may cause a serious inflammatory reaction. In addition, research has shown that allogeneic giant cells cluster near particles with uneven surfaces, and therefore the smoothness and uniformity of the individual microspheres minimize the inflammatory response.^69^ Thus, microspheres with diameters ranging from 20 to 50 μm appear to be excellent for cutaneous injections. Microspheres in this distribution are large enough to avoid phagocytosis, while remaining tiny enough to be effortlessly delivered through a fine 26G or 30G needle with no need for considerable force. Gold *et al.* also found that the diameter of PMMA microspheres between 30–50 μm was optimal for maximizing the surface area exposed to autologous collagen.^61^

Strikingly different from HA, the key components of these injectable fillers are relatively low hydrophilicity. Thus, these microspheres easily deposit when mixed with a solution with low viscosity, such as water and PBS buffer solution. However, a carrier solution with excessive viscosity will increase the injection force for extrusion through a needle. Thus, it appears that the appropriate viscosity of the carrier solution is critical in ensuing the uniform dispersion of microspheres, which enables tissue formation in the interlayer. Besides, the viscosity of the carrier gel, in which the microspheres are uniformly embedded, prevents clumping of the particles during the formation of the host tissue matrix.^67^Table 5 summaries the representative injectable fillers approved in the United States.

**Table 5:** Regenerative injectable fillers approved in the United States

Products	Manufacturer	Time of approval by FDA	Raw material	Diameter (μm)	Base solution	Lidocaine	Needle	Skin test	Indications	Storage (°C)	Duration (years)
Sculptra	Galderma	2004	PLLA microspheres	40–63	CMC, mannitol	Yes	25G	No	Injection into deep dermis for correction of moderate to severe nasolabial folds and other wrinkles/folds in adults with normal immunogenicity	25	1–2
AestheFill	Gegen Biotech	1984	PDLLA microspheres	30–70	CMC	Yes	25G	No	Injection into deep dermis for correction of moderate to severe nasolabial folds and other wrinkles/folds	25	2–3
Ellanse	Sinclair	2009 (CE)	PCL microspheres	25–50	CMC	Yes	27G	No	Subdermal implantation in the face for the lasting correction of wrinkles and facial aging signs or conditions	15–25	1–4
Artecoll	Suneva Medical Inc.	2006	PMMA microspheres	30–50	Bovine collagen solution	Yes	26G	Yes	Injection into the dermal-subcutaneous correction of facial wrinkles/folds and filling of soft tissues	2–8	5–10
Radiesse	Merz Aesthetics	2006	CaHA microspheres	25–45	Sterile water, glycerin, CMC	Yes	26G	No	Injected into the deep dermal subcutaneous for correction of moderate to severe nasolabial folds and wrinkles/folds	25	1–2

**Fig. 12:**
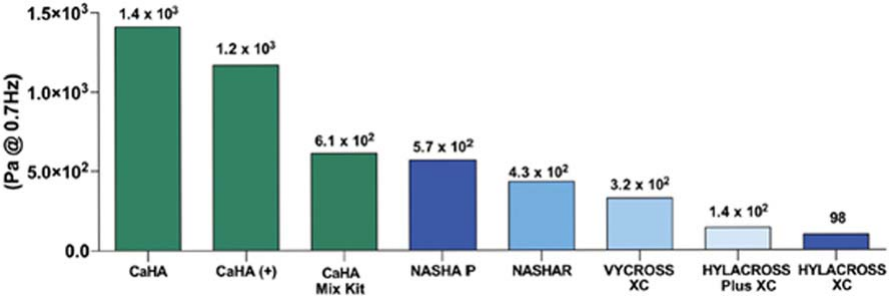
Elastic modulus (*G*′) of calcium hydroxylapatite (CaHA) in 3 formulations: CaHA alone, CaHA with integral lidocaine, and CaHA in a mix kit and 5 commercially available hyaluronic acid (HA) dermal fillers. This figure has been adapted from ref. 56 with permission from Lorenc, Copyright 2018.

Similar to HA fillers, understanding the rheological characteristics of biomaterial microspheres is critical because they can impact the filler performance. However, the rheological features of regeneration fillers are less investigated compared to that of HA fillers. Lorenc *et al.* investigated the lift capacity, deformation resistance, tissue integration and physicochemical properties of different HA fillers and CaHA using three animal models^56^ (Fig. 12). CaHA outperformed the HA fillers in terms of *G*′, immediate resistance to deformation, and sustained cohesivity at all time points. However, the larger *G*′ associated with CaHA did not always imply a stronger lift capacity compared to the HA fillers. The dramatic difference in *G*′ and viscosity between CaHA and HAs shows that they may be regarded complementary rather than competing.^70^

## Others

Outline/evolution is a copolymer of diallyldimethylammonium chloride and acrylamide that has been partially crosslinked by *N*,*N*′-methylenebisacrylamide and PVA hydrogel microspheres (5–40 μm). Because the copolymer and PVA have a positive charge, negatively charged tissue molecules such as hyaluronic acid and other amino acids glucan are drawn to them. Two months later, negatively charged tissue molecules progressively enter the implant and produce soft spongy material to improve the face contour.

Aquamid is a biocompatible, non-absorbable hydrogel made by polymerizing acrylamide monomers with *N*,*N*′-methylenebisacrylamide. Aquamid is composed of 2.5% crosslinked polyacrylamide and 97.5% water, which has been widely used for the treatment of different rhytids, facial contouring and correction, and its efficacy can last for more than 1 year. In 37 cases, the most common adverse effects were erythema, bruising, swelling, itching, and discomfort. A modest color change at the injection site and one incidence of neutropenia were among the unusual adverse events identified.^71^

## Perspectives

Besides the products described above that have been approved by the FDA, CE or NMPA, many modification strategies are being applied to reduce the complication rate and improve the properties of fillers, and new diverse materials are being researched to deliver ideal tissue regeneration.

The modification of molecular structure can avoid their shortcomings. Commercial HA-based fillers are made using crosslinking agents such as DVS and BDDE, which increase the toxic risk. To avoid the need for harmful chemical crosslinkers, Hong *et al.* modified an HA derivative with catechol groups that may self-crosslink *via* self-oxidation.^72^ Hong *et al.* used less toxic vitamin B2 derivatives as photo-initiators to introduce tyramine into HA to impart photo-crosslinking ability.^73^ To further extend the duration, amino acids were grafted onto HA to significantly reduce its enzymatic degradation.^74^ Schanté *et al.* demonstrated that amino acid-modified HA derivatives are good materials for biomedical applications, particularly HA-tyrosine, which also exhibited increased resistance to enzymatic digestion in a variety of amino-acid modified HA hydrogels.^75^ Because of the super-hydrophobicity of PLLA and PCL, their microspheres are different to mix uniformly in carrier solution, and hence polyethylene glycol (PEG) was introduced to increase the water solubility.^76^ Steinman *et al.* prepared a triblock copolymer of PCL-PEG-PCL with different molecular weights of PEG to increase the water solubility and showed its potential as dermal fillers.^77^ Cui *et al.* synthesized hydrogels composed of PLA-PEG-PLA copolymer with good water-solubility, which can be used in tissue engineering.^78^ CureWhite, a PLLA-PEG microsphere suspension in crosslinked HA hydrogels approved by NMPA in 2021, is indicated for the correction of moderate to severe nasolabial folds and wrinkles.

Besides the above-mentioned biomaterials, researchers are always exploiting new materials. Lee *et al.* suggested that autologous platelet-rich plasma (PRP) plays an essential role in increasing collagen expression, matrix remodeling proteins, fibroblast proliferation and differentiation into myofibroblasts.^79,80^ Kang *et al.* identified keratin-fibrinogen hydrogels as potential filler materials to accelerate tissue regeneration.^81^ Choi *et al.* created injectable and physical hydrogels by mixing levan with Pluronic and CMC, demonstrating the potential of levan as a novel material for dermal fillers.^82^ Kim *et al.* fabricated an HA-PN (hyaluronic acid-polynucleotide) complex filler by combining an HA-based filler with PN solution, which may promote fibroblast proliferation, volume expansion and skin regeneration.^83^

**Fig. 13:**
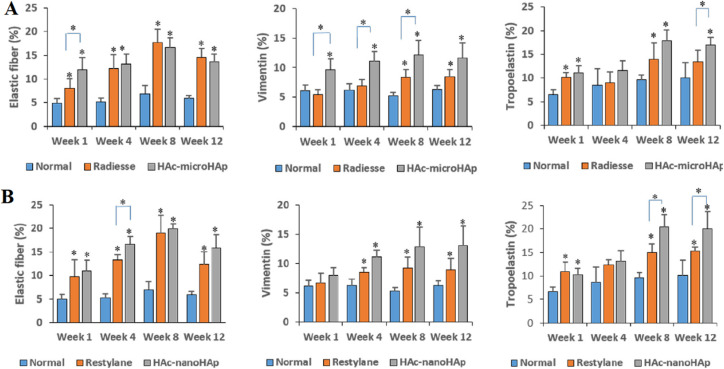
Collagen and elastic fiber formation in the Radiesse (A), Restylane (B) and HA-Hap composite filler. This figure has been adapted from ref. 84 with permission from Fan, copyright 2019.

The combination of materials may unite the unique properties of individual materials. Fan *et al.* fabricated a hyaluronic acid-hydroxyapatite composite hydrogel and proved the significant improvements in volumetric maintenance compared with the pure HA filler (Fig. 13A). The TGF-β/Samd pathway stimulated greater collagen and elastic fiber regeneration in the composite filler than in other available pure fillers, such as Radiesse and Restylane (Fig. 13B).^84^ Domouny *et al.* prepared an injectable dermal filler hydrogel made of a ternary combination of a polyanion and polyampholyte, hyaluronic acid (HA) and gelatin, linked by cationic cellulose nanocrystals (cCNCs).^85^

According to the trend of development, tissue engineering products have commercial potential to take a major market share in the future.^86,87^ Scaffolds and seed cells, as the major components of tissue engineering, are critical for the development of a tissue engineering product.^88^ Eça *et al.* investigated the safety and efficacy of intradermal injections of cultured autologous fibroblasts into forehead wrinkles, the perioral and paranasal areas and proved the safety and considerable improvement in periorbital skin flaccidity in five patients.^89^ Rhee *et al.* developed a long-term filler system comprised of a blend of HA filler and live human mesenchymal cells to maintain the impact tissue augmentation.^90^ Huang *et al.* demonstrated that human adipose stem cells proliferated and further differentiated into adipose tissue in HA hydrogels *in vitro*, retaining the potential of tissue filler.^91^ Zhao *et al.* reported the development of an injectable hydrogel derived from human acellular adipose tissue that may trigger the generation of human adipose stem cells (HASCs).^92^ These filler products may not only physically fill or cause a host response, but may also regenerate normal human soft tissue and further delay the natural aging processes.

Currently, anesthetics such as lidocaine are widely used with dermal fillers to minimize the discomfort during injection, but more drugs may be added in the future to provide other functions. Filler stimulation at the implant site should be evaluated both positively and negatively. Thus, by modulating and adjusting the stimulation, such as enhancing or inhibiting the growth of fibers with different drugs, the filler–drug combination may better match the proper host tissue responses. Fan *et al.* discovered that combining PLA microspheres and PEG-PCL-PEG micelles with dexamethasone may boost collagen production.^93^ Olmo *et al.* loaded the antibiotics cefuroxime (CFX), tetracycline (TCN), and amoxicillin (AMX) as well as the anti-inflammatory drug acetylsalicylic acid (ASA) into a crosslinked HA hydrogel to promote the antibacterial and anti-inflammatory response.^94^

Combination therapies, in addition to combining fillers with drugs and medications enable an appropriate response to the multifactorial process of aging, which involves structural changes in all anatomical layers and dynamic interactions among these tissues.^95–97^ Multiple aims such as relaxing, volumisation, volume relocation, reshaping, resurfacing, or tightening can be achieved using the complete, three-dimensional and multi-layered strategy, which combines multiple agents and procedures.^98,99^ Melo concluded that combination therapies have additive or even synergistic benefits, resulting in better and longer-lasting therapeutic outcomes than single agent- or single technique-based protocols, with no clinical evidence of increased incidence or severity of side events^100^ (Table 6).

**Table 6:** The representative combinations of treatment^a^

Face	Neck
Randomized controlled trial (*n* patients)	
HA filler + RF *vs.* HA filler (*n* = 10)	
BoNTx + HA filler *vs.* BoNTx (*n* = 20)	
BoNTx + HA filler *vs.* BoNTx + HA filler + cosmetic treatment (*n* = 20)	
BoNTx + HA filler *vs.* BoNTx *vs.* HA filler (*n* = 90)	

Non-randomized studies and case reports (*n* participants)	
HA filler + radiofrequency (*n* = 1)	BoNTx + HA filler + MFU-V (or CaHA) (*n* = 101)
BoNTx + HA filler + laser resurfacing (*n* = 1)	BoNTx + HA filler + IFU (intensity focused ultrasound) (*n* = 12)
BoNTx + CaHA + HA filler + PLLA (*n* = 2)	CaHA + MFU-V (micro-focused ultrasound) (*n* = 47)
BoNTx + CaHA + HA filler + MFU-V (micro-focused ultrasound) (*n* = 101)	BoNTx + CaHA + HA filler + MFU-V (micro-focused ultrasound) (*n* = 101)
BoNTx + HA filler (*n* = 60)	
Bimatoprost + BoNTx + HA filler (*n* = 116)	

Reviews	
BoNTx + HA filler + various EBDs (laser, IPL, MFUS, FMR)	Various techniques
BoNTx + HA filler + IPL (intense pulsed light) + lasers + radiofrequency	

a This table has been adapted from ref. 100 with permission from Melo, Copyright 2020.

## Conclusions

For decades, patients and clinicians have been attracted to the use of injectable fillers for soft tissue augmentation. Multiple types of injectable fillers have been invented, optimized and commercialized, while others have vanished from the market for some specific reasons. In this review, we summarized the majority of currently available injectable fillers, highlighted critical indicators, and investigated their function mechanisms. The critical parameters including concentration, rheology, microsphere size, and viscosity are the key differences between these products and result in unique actions. Given the current limits, innovative solutions for optimal tissue augmentation generation must be studied to suit the increasingly diverse demands by patients.

## Author contributions

Conceptualization, Jiahong Guo; writing – original draft preparation, Jiahong Guo; writing – review & editing, Jiahong Guo, Wei Fang, Feifei Wang; supervision, Wei Fang, Feifei Wang.

## Conflicts of interest

There are no conflicts to declare.

## Supplementary Material
